# Genome sequence of *Ensifer medicae* strain WSM1115; an acid-tolerant *Medicago*-nodulating microsymbiont from Samothraki, Greece

**DOI:** 10.4056/sigs.4938652

**Published:** 2013-12-31

**Authors:** Wayne Reeve, Ross Ballard, John Howieson, Elizabeth Drew, Rui Tian, Lambert Bräu, Christine Munk, Karen Davenport, Patrick Chain, Lynne Goodwin, Ioanna Pagani, Marcel Huntemann, Konstantinos Mavrommatis, Amrita Pati, Victor Markowitz, Natalia Ivanova, Tanja Woyke, Nikos Kyrpides

**Affiliations:** 1 Centre for Rhizobium Studies, Murdoch University, Western Australia, Australia; 2 South Australian Research and Development Institute, Urrbrae, South Australia, Australia; 3 School of Life and Environmental Sciences, Deakin University, Victoria, Australia; 4 Los Alamos National Laboratory, Bioscience Division, Los Alamos, New Mexico, USA; 5 DOE Joint Genome Institute, Walnut Creek, California, USA; 6 Biological Data Management and Technology Center, Lawrence Berkeley National Laboratory, Berkeley, California, USA

**Keywords:** root-nodule bacteria, nitrogen fixation, rhizobia, *Alphaproteobacteria*

## Abstract

*Ensifer medicae* strain WSM1115 forms effective nitrogen fixing symbioses with a range of annual *Medicago* species and is used in commercial inoculants in Australia. WSM1115 is an aerobic, motile, Gram-negative, non-spore-forming rod. It was isolated from a nodule recovered from the root of burr medic (*Medicago polymorpha*) collected on the Greek Island of Samothraki. WSM1115 has a broad host range for nodulation and N_2_ fixation capacity within the genus *Medicago*, although this does not extend to all medic species. WSM1115 is considered saprophytically competent in moderately acid soils (pH(CaCl_2_) 5.0), but it has failed to persist at field sites where soil salinity exceeded 10 ECe (dS/m). Here we describe the features of *E. medicae* strain WSM1115, together with genome sequence information and its annotation. The 6,861,065 bp high-quality-draft genome is arranged into 7 scaffolds of 28 contigs, contains 6,789 protein-coding genes and 83 RNA-only encoding genes, and is one of 100 rhizobial genomes sequenced as part of the DOE Joint Genome Institute 2010 Genomic Encyclopedia for *Bacteria* and *Archaea*-Root Nodule *Bacteria* (GEBA-RNB) project.

## Introduction

The genus *Medicago* comprises 87 species of annual and perennial legumes, including some that were formerly recognized as *Trigonella* and *Melilotus* species [1]. A small number of annual *Medicago* species that have been domesticated are grown extensively in the sheep-wheat zone of southern Australia, particularly where pasture regeneration after a cropping phase is desirable. Annual *Medicago* species are grown on more than 20 M ha [2] and are particularly valued for their contribution to farming systems, in which *Medicago* fix around 25 kg of N per tonne of legume dry matter produced [3].

*Medicago* are nodulated by two species of root nodule bacteria (*Ensifer medicae* and *Ensifer meliloti*) that are recognized as being distinct based on their different nodulation and N_2_ fixation phenotypes in host interaction studies and more detailed analyses of their genetics [4,5].

*Ensifer medicae* strain WSM1115 is used in Australia to produce commercial peat cultures (referred to as Group AM inoculants) for the inoculation of several species of annual *Medicago* (predominantly *M. truncatula*, *M. polymorpha*, *M. scutellata*, *M. sphaerocarpus*, *M. murex*, *M. rugosa* and *M. orbicularis*). WSM1115 has been used commercially since 2002 [6], when it replaced strain WSM688. WSM1115 was isolated from a nodule from the roots of burr medic (*Medicago polymorpha*) collected by Prof. John Howieson (Murdoch University, Australia) on the island of Samothraki, Greece.

WSM1115 was selected for use in commercial inoculants having demonstrated good N_2_-fixation capacity with the relevant medic hosts and adequate saprophytic competence in moderately acidic soil (pH(CaCl_2_) 5).

Saprophytic competence in acidic soils is a requirement of strains used to inoculate *Medicago* because several species (*M. murex*, *M. sphaerocarpus* and *M. polymorpha*) are recommended and sown into soils below pH(CaCl_2_) 5.5, a level that is known to limit both survival of medic rhizobia and nodulation processes [7-10]. Useful variation in saprophytic competence occurs between strains of medic rhizobia [9] and valuable insights into the mechanisms that confer acidity tolerance have been provided by studies using strain WSM419 [11], which has been recently sequenced [12]. However, the complex nature of soil adaptation means that *in-situ* field studies still provide the most reliable means of selecting an inoculant strain and were used to select WSM1115 for commercial use. In a cross row experiment comparing 15 strains on acidic sand (pH(CaCl_2_) 5.0; Dowerin, West Australia), the nodulation of plants inoculated with WSM1115 was equal to or better than that of the other strains. This translated to better plant shoot weights, which were similar to those of plants inoculated with WSM688 (the incumbent inoculant strain at time of testing) and 48% greater when compared to former inoculant strain CC169 (J. G. Howieson unpublished data).

The nitrogen fixation capacity (effectiveness) of *Medicago* symbioses is characterized by strong interactions between the strain of rhizobia and species of *Medicago* [13-16]. Hence, the ability to form effective symbiosis with the species recommended for inoculation is an important consideration in inoculant strain selection. WSM1115 satisfies this requirement. In greenhouse tests it formed effective symbiosis with 16 genotypes of *Medicago* and overall produced 48% more shoot dry matter compared to plants inoculated with WSM688, the strain that it replaced (R.A. Ballard and N. Charman, unpublished data).

A limitation of strain WSM1115 is its poor persistence in moderately saline soils (e.g. where summer salinity levels exceed 10 ECe (dS/m)). Poor nodulation of regenerating pasture was first noted in 2004 during the field evaluation and domestication of the salt tolerant annual pasture legume messina (*Melilotus siculus* syn. *Melilotus messanensis*). Subsequent studies [17] confirmed that although WSM1115 was able to nodulate and form effective symbiosis with messina, it did not persist as well as other strains (e.g. SRDI554) through the summer months when salinity levels increased.

Here we present a preliminary description of the general features of *Ensifer medicae* strain WSM1115 together with its genome sequence and annotation.

## Classification and features

**Figure 1:**
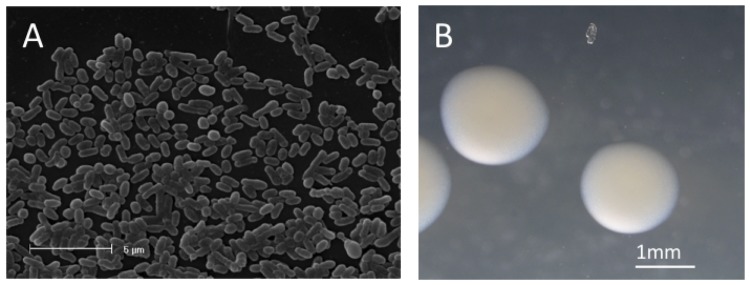
Images of *Ensifer medicae* strain WSM1115 using (A) scanning electron microscopy and (B) light microscopy to show the colony morphology on a solid medium.

*Ensifer medicae* strain WSM1115 is a motile, non-sporulating, non-encapsulated, Gram-negative rod in the order *Rhizobiales* of the class *Alphaproteobacteria*. The rod-shaped form varies in size with dimensions of approximately 0.5 μm in width and 1.0 μm in length (Figure 1A). It is fast growing, forming colonies within 3-4 days when grown on TY [18] or half strength Lupin Agar (½LA) [19] at 28°C. Colonies on ½LA are opaque, slightly domed and moderately mucoid with smooth margins (Figure 1B).

**Figure 2:**
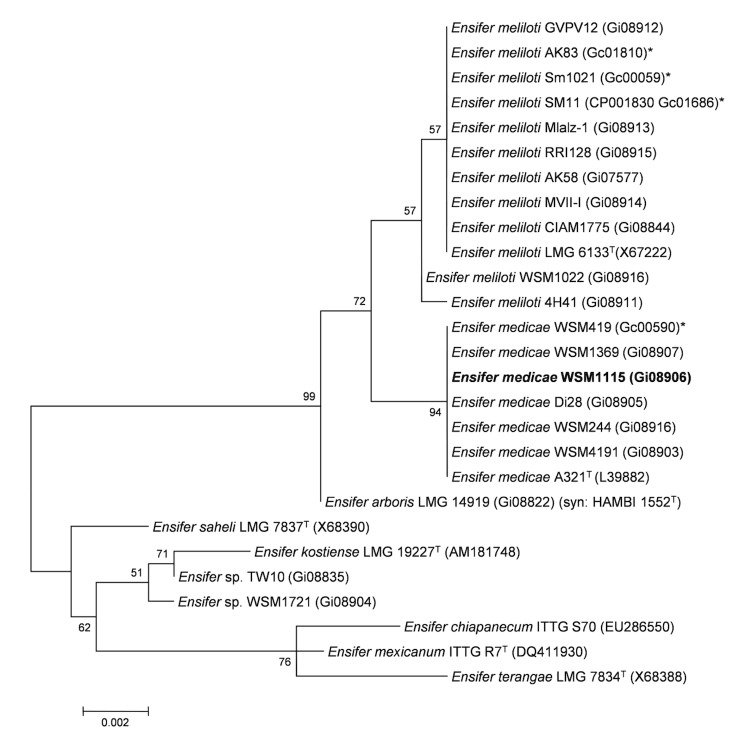
Phylogenetic tree showing the relationship of *Ensifer medicae* WSM1115 (shown in bold print) to other *Ensifer* spp. in the order *Rhizobiales* based on aligned sequences of the 16S rRNA gene (1,290 bp internal region). All sites were informative and there were no gap-containing sites. Phylogenetic analyses were performed using MEGA, version 5 [33]. The tree was built using the Maximum-Likelihood method with the General Time Reversible model [34]. Bootstrap analysis [35] with 500 replicates was performed to assess the support of the clusters. Type strains are indicated with a superscript T. Brackets after the strain name contain a DNA database accession number and/or a GOLD ID (beginning with the prefix G) for a sequencing project registered in GOLD [32]. Published genomes are indicated with an asterisk.

Minimum Information about the Genome Sequence (MIGS) is provided in Table 1. Figure 2 shows the phylogenetic neighborhood of *Ensifer medicae* strain WSM1115 in a 16S rRNA gene sequence based tree. This strain has 100% sequence identity (1,366/1,366 bp) at the 16S rRNA sequence level to the fully sequenced *Ensifer medicae* strain WSM419 [12] and 99% 16S rRNA sequence (1362/1366 bp) identity to the fully sequenced *E. meliloti* Sm1021 [36].

**Table 1:** Classification and general features of *Ensifer medicae* strain WSM1115 according to the MIGS recommendations [20]

**MIGS ID**	**Property**	**Term**	**Evidence code**
	Current classification	Domain *Bacteria*	TAS [21]
		Phylum *Proteobacteria*	TAS [22]
		Class *Alphaproteobacteria*	TAS [23,24]
		Order *Rhizobiales*	TAS [22,25]
		Family *Rhizobiaceae*	TAS [26,27]
		Genus *Ensifer*	TAS [28-30]
		Species *Ensifer medicae*	TAS [29]
		Strain WSM1115	
	Gram stain	Negative	IDA
	Cell shape	Rod	IDA
	Motility	Motile	IDA
	Sporulation	Non-sporulating	NAS
	Temperature range	Mesophile	NAS
	Optimum temperature	28°C	NAS
	Salinity	Non-halophile	NAS
MIGS-22	Oxygen requirement	Aerobic	IDA
	Carbon source	Varied	NAS
	Energy source	Chemoorganotroph	NAS
MIGS-6	Habitat	Soil, root nodule, on host	IDA
MIGS-15	Biotic relationship	Free living, symbiotic	IDA
MIGS-14	Pathogenicity	Non-pathogenic	IDA
	Biosafety level	1	TAS [31]
	Isolation	Root nodule	IDA
MIGS-4	Geographic location	Samothraki, Greece	IDA
MIGS-5	Time of sample collection	May, 1987	IDA
MIGS-4.1	Latitude	40.4900	IDA
MIGS-4.2	Longitude	25.6500	IDA
MIGS-4.3	Depth	<10 cm	IDA
MIGS-4.4	Altitude	325 m	IDA

Evidence codes – IDA: Inferred from Direct Assay; TAS: Traceable Author Statement (i.e., a direct report exists in the literature); NAS: Non-traceable Author Statement (i.e., not directly observed for the living, isolated sample, but based on a generally accepted property for the species, or anecdotal evidence). These evidence codes are from the Gene Ontology project [32].

### Symbiotaxonomy

*Ensifer medicae* strain WSM1115 forms nodules (Nod+) and fixes N_2_ (Fix+) with a range of annual and perennial *Medicago* species and *Melilotus* species (Table 2). Levels of N_2_ fixation in combination with *Medicago littoralis* is suboptimal, that species generally forming more effective associations with strains of *Ensifer meliloti* including strain RRI128 [38]. The level of N_2_ fixation with *Melilotus albus* is also noted as positive, but has been observed to vary markedly with different plant accessions.

**Table 2:** Compatibility of *Ensifer medicae* WSM1115 with various *Medicago* and allied genera for nodulation (Nod) and N_2_-fixation (Fix)

**Species Name**	**Cultivar or line**	**Common Name**	**Growth Type**	**Nod**	**Fix**	**Reference**
*M. polymorpha*	Santiago/Cavalier/Scimitar	Burr	Annual	+	+	IDA
*M. truncatula.*	Caliph/Jester	Barrel	Annual	+	+	IDA
*M. murex*	Zodiac	Murex	Annual	+	+	IDA
*M. sphaerocarpus*	Orion	Sphere	Annual	+	+	IDA
*M. scutellata*	Sava/Silver/Essex	Snail	Annual	+	+	IDA
*M. rugosa*	Paraponto	Gama	Annual	+	+	IDA
*M. littoralis*	Herald/Harbinger	Strand	Annual	+	Poor	IDA
*M. orbicularis*	Estes	Button	Annual	+	+	[15]
*M. rigiduloides*	Accession PI 227850	Rigid	Annual	+(w)	-	[15]
*M. rigidula*	Accession PI 495552	Tifton	Annual	+(w)	-	[15]
*M. arabica*	Local ecotype	Spotted	Annual	+	+	[15]
*M. minima*	Devine	Woolly burr	Annual	+	+	[15]
*M. sativa*	SARDI Ten	Lucerne	Perennial	+	+	IDA
*M. lupulina*	‘BEBLK’	Black	Perennial	+	+	[15]
*Melilotus siculus*	Accessions SA40006 & 39909	Messina	Annual	+	+	[17]
*Melilotus albus*	various accessions	Bokhara clover	Biennial	+	+	IDA

(w) indicates nodules present were white.

IDA: Inferred from Direct Assay from the Gene Ontology project [37].

## Genome sequencing and annotation information

### Genome project history

This organism was selected for sequencing on the basis of its environmental and agricultural relevance to issues in global carbon cycling, alternative energy production, and biogeochemical importance, and is part of the Community Sequencing Program at the U.S. Department of Energy, Joint Genome Institute (JGI) for projects of relevance to agency missions. The genome project is deposited in the Genomes OnLine Database [32] and a high-quality-draft genome sequence in IMG/GEBA. Sequencing, finishing and annotation were performed by the JGI. A summary of the project information is shown in Table 3.

**Table 3:** Genome sequencing project information for *Ensifer medicae* strain WSM1115

**MIGS ID**	**Property**	**Term**
MIGS-31	Finishing quality	Permanent high quality draft
MIGS-28	Libraries used	2× Illumina libraries; Std short PE & CLIP long PE
MIGS-29	Sequencing platforms	Illumina HiSeq 2000
MIGS-31.2	Sequencing coverage	530× Illumina
MIGS-30	Assemblers	with Allpaths, version 38445, Velvet 1.1.05, phrap 4.24
MIGS-32	Gene calling methods	Prodigal 1.4, GenePRIMP
	Genbank ID	AQZC01000000
	Genbank Date of Release	April 22, 2013
	GOLD ID	Gi08906
	NCBI project ID	74391
	Database: IMG-GEBA	2512875026
	Project relevance	Symbiotic N_2_ fixation, agriculture

### Growth conditions and DNA isolation

*Ensifer medicae* strain WSM1115 was cultured to mid logarithmic phase in 60 ml of TY rich medium on a gyratory shaker at 28°C [39]. DNA was isolated from the cells using a CTAB (Cetyl trimethyl ammonium bromide) bacterial genomic DNA isolation method [40].

### Genome sequencing and assembly

The genome of *Ensifer medicae* strain WSM1115 was sequenced at the Joint Genome Institute (JGI) using Illumina [41] data. An Illumina standard paired-end library with a minimum insert size of 270 bp was used to generate 23,080,558 reads totaling 3,462 Mbp and an Illumina CLIP paired-end library with an average insert size of 9,584 + 2,493 bp was used to generate 2,163,668 reads totaling 324 Mbp of Illumina data (unpublished, Feng Chen).

All general aspects of library construction and sequencing performed at the JGI can be found at the JGI user home [40]. The initial draft assembly contained 57 contigs in 11 scaffolds. The initial draft data was assembled with Allpaths, version 38445, and the consensus was computationally shredded into 10 Kbp overlapping fake reads (shreds). The Illumina draft data was also assembled with Velvet, version 1.1.05 [42], and the consensus sequences were computationally shredded into 1.5 Kbp overlapping fake reads (shreds). The Illumina draft data was assembled again with Velvet using the shreds from the first Velvet assembly to guide the next assembly. The consensus from the second VELVET assembly was shredded into 1.5 Kbp overlapping fake reads. The fake reads from the Allpaths assembly and both Velvet assemblies and a subset of the Illumina CLIP paired-end reads were assembled using parallel phrap, version 4.24 (High Performance Software, LLC). Possible mis-assemblies were corrected with manual editing in Consed [43-45]. Gap closure was accomplished using repeat resolution software (Wei Gu, unpublished), and sequencing of bridging PCR fragments. The estimated total size of the genome is 6.9 Mbp and the final assembly is based on 3,654 Mbp of Illumina draft data, which provides an average 530× coverage of the genome.

### Genome annotation

Genes were identified using Prodigal [46] as part of the Oak Ridge National Laboratory genome annotation pipeline, followed by a round of manual curation using the JGI GenePRIMP pipeline [47]. The predicted CDSs were translated and used to search the National Center for Biotechnology Information (NCBI) nonredundant database, UniProt, TIGRFam, Pfam, PRIAM, KEGG, COG, and InterPro databases. These data sources were combined to assert a product description for each predicted protein. Non-coding genes and miscellaneous features were predicted using tRNAscan-SE [48], RNAMMer [49], Rfam [50], TMHMM [51], and SignalP [52]. Additional gene prediction analyses and functional annotation were performed within the Integrated Microbial Genomes (IMG-ER) platform [53].

## Genome properties

The genome is 6,861,065 nucleotides with 61.16% GC content (Table 4) and comprised of 7 scaffolds (Figures 3a,3b,3c,3d,3e,3f and Figure 3g) From a total of 6,872 genes, 6,789 were protein encoding and 83 RNA only encoding genes. The majority of genes (76.25%) were assigned a putative function whilst the remaining genes were annotated as hypothetical. The distribution of genes into COGs functional categories is presented in Table 5.

**Table 4:** Genome Statistics for *Ensifer medicae* strain WSM1115

**Attribute**	**Value**	**% of Total**
Genome size (bp)	6,861,065	100.00
DNA coding region (bp)	5,918,651	86.26
DNA G+C content (bp)	4,196,062	61.16
Number of scaffolds	7	
Number of contigs	28	
Total gene	6,872	100.00
RNA genes	83	1.21
rRNA operons	3	0.04
Protein-coding genes	6,789	98.79
Genes with function prediction	5,240	76.25
Genes assigned to COGs	5,168	75.20
Genes assigned Pfam domains	5,424	78.93
Genes with signal peptides	571	8.31
Genes coding membrane proteins	1,483	21.58
CRISPR repeats	0	

**Figure 3a:**
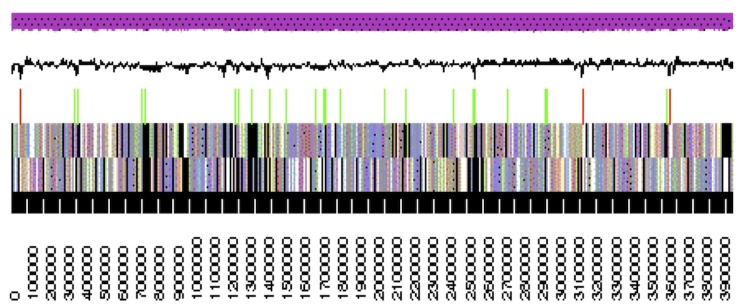
Graphical maps of SinmedDRAFT_Scaffold1.2 of the *Ensifer medicae* strain WSM1115 genome sequence. From bottom to the top of each scaffold: Genes on forward strand (color by COG categories as denoted by the IMG platform), Genes on reverse strand (color by COG categories), RNA genes (tRNAs green, sRNAs red, other RNAs black), GC content, GC skew.

**Figure 3b:**
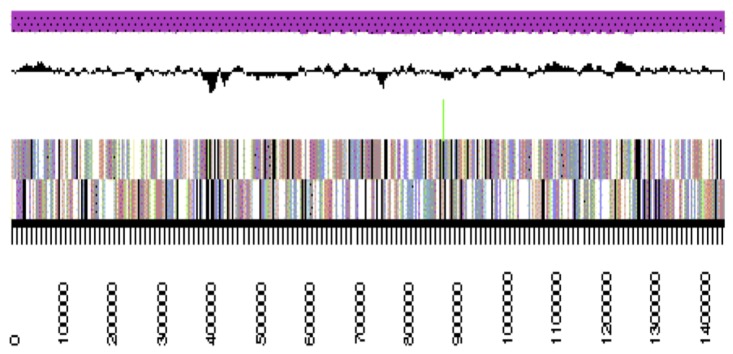
Graphical maps of SinmedDRAFT_Scaffold2.1 of the *Ensifer medicae* strain WSM1115 genome sequence. From bottom to the top of each scaffold: Genes on forward strand (color by COG categories as denoted by the IMG platform), Genes on reverse strand (color by COG categories), RNA genes (tRNAs green, sRNAs red, other RNAs black), GC content, GC skew.

**Figure 3c:**
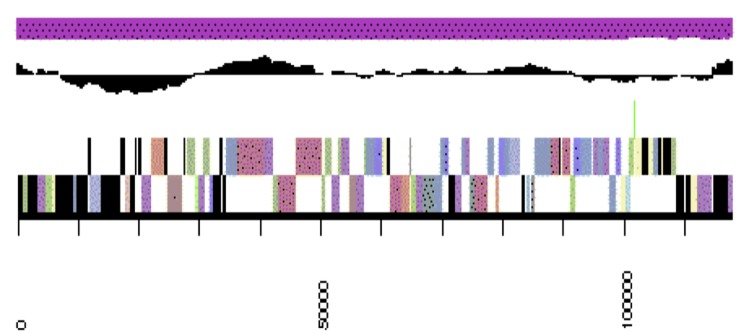
Graphical maps of SinmedDRAFT_Scaffold5.3 of the *Ensifer medicae* strain WSM1115 genome sequence. From bottom to the top of each scaffold: Genes on forward strand (color by COG categories as denoted by the IMG platform), Genes on reverse strand (color by COG categories), RNA genes (tRNAs green, sRNAs red, other RNAs black), GC content, GC skew.

**Figure 3d:**
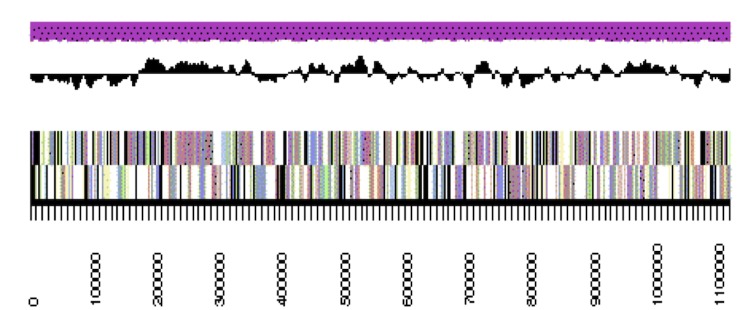
Graphical maps of SinmedDRAFT_Scaffold3.7 of the *Ensifer medicae* strain WSM1115 genome sequence. From bottom to the top of each scaffold: Genes on forward strand (color by COG categories as denoted by the IMG platform), Genes on reverse strand (color by COG categories), RNA genes (tRNAs green, sRNAs red, other RNAs black), GC content, GC skew.

**Figure 3e:**
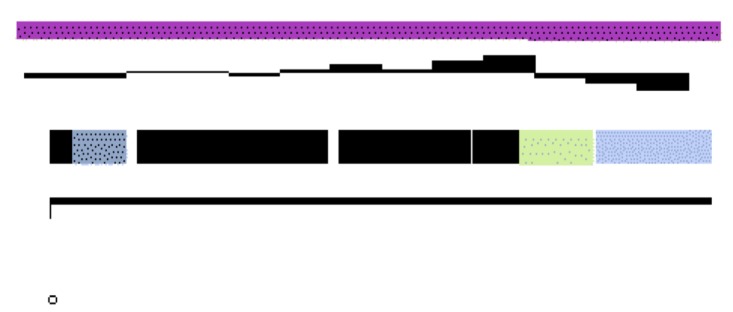
Graphical maps of SinmedDRAFT_Scaffold6.5 of the *Ensifer medicae* strain WSM1115 genome sequence. From bottom to the top of each scaffold: Genes on forward strand (color by COG categories as denoted by the IMG platform), Genes on reverse strand (color by COG categories), RNA genes (tRNAs green, sRNAs red, other RNAs black), GC content, GC skew.

**Figure 3f:**
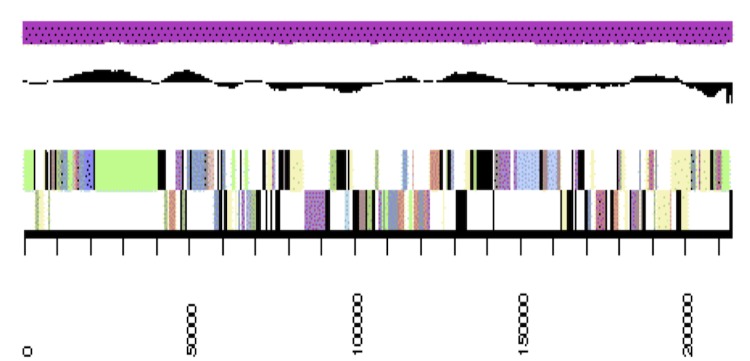
Graphical maps of SinmedDRAFT_Scaffold4.6 of the *Ensifer medicae* strain WSM1115 genome sequence. From bottom to the top of each scaffold: Genes on forward strand (color by COG categories as denoted by the IMG platform), Genes on reverse strand (color by COG categories), RNA genes (tRNAs green, sRNAs red, other RNAs black), GC content, GC skew.

**Figure 3g:**
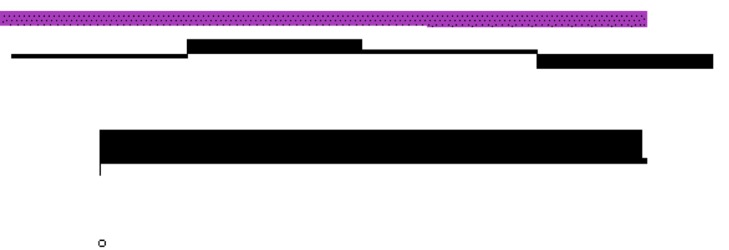
Graphical maps of SinmedDRAFT_Scaffold7.4 of the *Ensifer medicae* strain WSM1115 genome sequence. From bottom to the top of each scaffold: Genes on forward strand (color by COG categories as denoted by the IMG platform), Genes on reverse strand (color by COG categories), RNA genes (tRNAs green, sRNAs red, other RNAs black), GC content, GC skew.

**Table 5:** Number of protein coding genes of *Ensifer medicae* strain WSM1115 associated with the general COG functional categories.

**Code**	**Value**	**%age**	**COG Category**
J	186	3.23	Translation, ribosomal structure and biogenesis
A	0	0.00	RNA processing and modification
K	527	9.16	Transcription
L	269	4.68	Replication, recombination and repair
B	3	0.05	Chromatin structure and dynamics
D	43	0.75	Cell cycle control, mitosis and meiosis
Y	0	0.00	Nuclear structure
V	55	0.96	Defense mechanisms
T	244	4.24	Signal transduction mechanisms
M	272	4.73	Cell wall/membrane biogenesis
N	68	1.18	Cell motility
Z	0	0.00	Cytoskeleton
W	1	0.02	Extracellular structures
U	112	1.95	Intracellular trafficking and secretion
O	195	3.39	Posttranslational modification, protein turnover, chaperones
C	335	5.82	Energy production conversion
G	575	10.00	Carbohydrate transport and metabolism
E	609	10.59	Amino acid transport metabolism
F	106	1.84	Nucleotide transport and metabolism
H	194	3.37	Coenzyme transport and metabolism
I	205	3.56	Lipid transport and metabolism
P	286	4.97	Inorganic ion transport and metabolism
Q	164	2.85	Secondary metabolite biosynthesis, transport and catabolism
R	726	12.62	General function prediction only
S	577	10.03	Function unknown
-	1,704	24.80	Not in COGS
-	5,752		Total
